# Mapping the ionosphere with millions of phones

**DOI:** 10.1038/s41586-024-08072-x

**Published:** 2024-11-13

**Authors:** Jamie Smith, Anton Kast, Anton Geraschenko, Y. Jade Morton, Michael P. Brenner, Frank van Diggelen, Brian P. Williams

**Affiliations:** 1 Google Research, Mountain View, CA USA; 2 Aerospace Engineering Sciences Department, University of Colorado Boulder, Boulder, CO USA; 3 School of Engineering and Applied Sciences, Harvard University, Cambridge, MA USA; 4 Google Platforms and Devices, Mountain View, CA USA

**Keywords:** Space physics, Atmospheric dynamics, Technology

## Abstract

The ionosphere is a layer of weakly ionized plasma bathed in Earth’s geomagnetic field extending about 50–1,500 kilometres above Earth^1^. The ionospheric total electron content varies in response to Earth’s space environment, interfering with Global Satellite Navigation System (GNSS) signals, resulting in one of the largest sources of error for position, navigation and timing services^2^. Networks of high-quality ground-based GNSS stations provide maps of ionospheric total electron content to correct these errors, but large spatiotemporal gaps in data from these stations mean that these maps may contain errors^3^. Here we demonstrate that a distributed network of noisy sensors—in the form of millions of Android phones—can fill in many of these gaps and double the measurement coverage, providing an accurate picture of the ionosphere in areas of the world underserved by conventional infrastructure. Using smartphone measurements, we resolve features such as plasma bubbles over India and South America, solar-storm-enhanced density over North America and a mid-latitude ionospheric trough over Europe. We also show that the resulting ionosphere maps can improve location accuracy, which is our primary aim. This work demonstrates the potential of using a large distributed network of smartphones as a powerful scientific instrument for monitoring Earth.

## Abstract (web summary)

Data from millions of smartphones are used to map the ionosphere in greater detail, leading to improved smartphone location accuracy, particularly in parts of the world with few monitoring stations.

## Main

There are billions of smartphones worldwide, each equipped with a powerful processor and a wide array of sensors. Although these sensors are generally of lower quality than those in conventional scientific instruments, the number and ubiquity of smartphones offer advantages over existing infrastructure in coverage and resolution. In this paper, we produce maps of the ionospheric total electron content (TEC) using satellite navigation signals from millions of smartphones equipped with dual-frequency Global Navigation Satellite System (GNSS) receivers.

The conditions in the ionosphere are dynamic: the electron density varies depending on the location, time, and solar and geomagnetic activities. Steep spatial gradients in ionospheric TEC often cause plasma density structures that disturb trans-ionospheric radio signals^4^. The ionospheric TEC refers to the number of free electrons contained in a column of unit cross-sectional area measured in TEC units (1 TECU = 10^16^ electrons per square metre). TEC is an important indicator of the state of the ionosphere and space weather. Global real-time ionospheric TEC maps are needed to produce space-weather operational products to serve a broad spectrum of civilian and security activities, including electric power distribution, aviation, satellite operations, navigation, precision agriculture and communications^5^. Current operational ionospheric and space-weather products provide limited coverage, spatial resolution and refresh rates, especially real-time products^6^.

Global Positioning System (GPS) and other GNSS receivers estimate distances to satellites by measuring the time for a radio signal to travel from a satellite to a receiver. Ionospheric TEC affects the propagation speed of radio waves, introducing significant errors in receivers’ calculations of the distance to satellites. This is one of the largest sources of geolocation error, and many GNSS receivers use a coarse spatiotemporal model of the ionospheric TEC to compensate for it. Most phones use an 8-parameter model, mitigating about 50% of the ionospheric error^7^. This simple model was developed in the early days of GPS and was optimized for the limited computation and bandwidth available at the time.

**Fig. 1:**
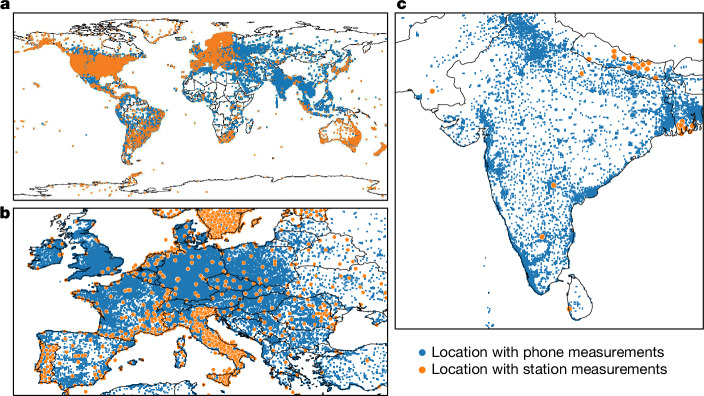
Geographical coverage of phones and monitoring stations. **a**–**c**, The 9,036 monitoring stations (orange dots) contribute to the Madrigal database, which consolidates dozens of global and regional station networks. The blue dots show approximately 100,000 locations where phone measurements are available. A location within a big city may have thousands of phones. The global map (**a**) shows that some parts of the world (such as the USA and Europe) are densely covered by monitoring stations. Zooming-in on Europe (**b**) shows that phones have even denser coverage. In India (**c**), the relative coverage of phones is greater still. Station locations were taken from the Madrigal database^23^. Locations with phones are from the present study. Source Data

Although ionospheric error presents a challenge for navigation, this same effect allows us to use a GNSS receiver to measure ionospheric TEC. The ionosphere causes a smaller delay on signals with a higher frequency. By measuring the discrepancy in travel time between signals of different frequencies, we can estimate the TEC along a line of sight between a dual-frequency GNSS receiver and a satellite. This approach has long been used with high-quality ground-based GNSS stations^8^, but these stations are expensive to install and maintain. Some regions are sparsely covered, leaving gaps in the picture of the ionosphere (Fig. 1, orange dots).

Using a distributed sensor network consisting of millions of mobile phones, we are able to resolve ionospheric phenomena with effectively double the measurement coverage. Although using low-cost receivers to improve the spatiotemporal resolution of TEC maps has been proposed before^9–15^, these efforts have not progressed past ideation, encountering significant organizational and technical challenges for ionosphere monitoring^16^. We show that a sufficient number of mobile phones offers a valuable resource for TEC mapping. Figure 1 contrasts the global distribution of monitoring stations with locations where Android phones generate dual-frequency GNSS measurements. Although both phones and monitoring stations have dense coverage in many regions, mobile phones provide unmatched coverage in Eastern Europe, India, South Asia, much of South America and parts of Africa.

We map the ionosphere using measurements from a population of Android devices that have location and relevant settings enabled and are using satellite signals to determine location. Android users can allow sensor data collection for improving location accuracy. Previous improvements have used measurements from users’ phones to enable indoor localization when satellite-based positioning is unavailable. Mapping the ionosphere with satellite measurements from phones will improve outdoor location accuracy by mitigating errors caused by inaccurate knowledge of the ionosphere. See Methods for more information on data collection.

Android’s GNSS application programming interface^17^ provides similar data to GNSS monitoring stations, including the satellite identifier, carrier frequency, and times of signal transmission and reception. However, mobile phones present a number of challenges. Antennas are much smaller, and GNSS hardware is less sophisticated than dedicated monitoring stations, leading to noisier measurements. Monitoring stations are stationary with a clear view of the sky, whereas mobile phones are often in users’ pockets or in urban canyons. Moreover, each individual phone has its own unique bias, determined by its hardware and various environmental factors. To achieve accurate results, we must mitigate the bias of each phone individually.

**Fig. 2:**
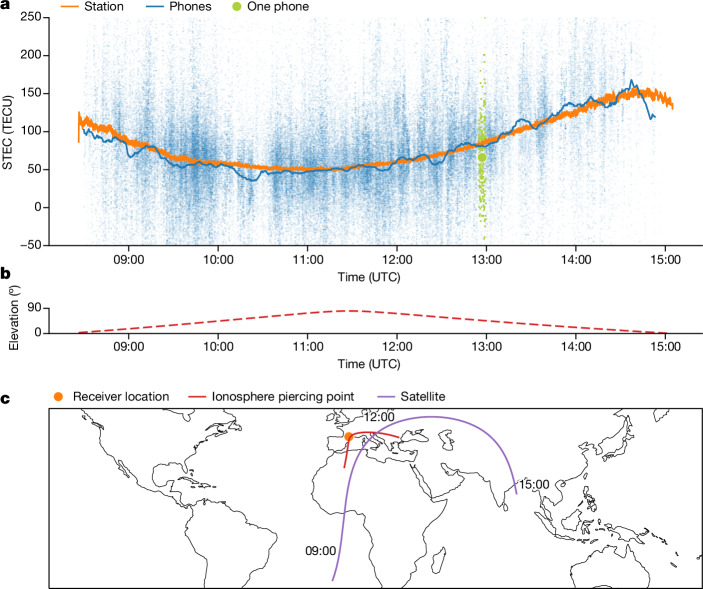
Comparison of phone and station measurements. Ionospheric TEC measurements along the path from a satellite to the ground by 1,011 phones and by a GNSS monitoring station (TLSE00FRA) in Toulouse, France (UTC + 1) on 5 November 2023. **a**, Comparison of measurements from a GNSS monitoring station with nearby phones over the same time period. Each blue point is a single TEC measurement to this satellite from a phone within 10 km of the monitoring station. Measurements from a single phone are shown in green to show the level of noise. The solid blue line shows the median of measurements from all phones in the area using a 10-minute sliding window. (The median is robust to infrequent outlier measurements beyond the axis limits.) The orange line shows the ionospheric TEC measured by monitoring station TLSE00FRA. **b**, Elevation angle to the satellite as it rises, passes the receiver antenna overhead and then sets. **c**, Map of the receiver location (Toulouse, France), the ground track of the satellite (GPS PRN 30 in purple) and the ionospheric piercing-point track of the GPS signal (red). The station measurements in **a** and the station location and satellite track in **b** and **c** are from International GNSS Service^35^ and were obtained via NASA Crustal Dynamics Data Information System^36^. All phone data are from the present study. Source Data

A GNSS receiver measures the ionosphere TEC along a line of sight from a satellite using the difference in measured travel times^18^, *t*_1_ and *t*_2_, for the satellite signal on two different frequencies, *f*_1_ and *f*_2_, respectively. The TEC along the path is approximated to first order in the frequencies by:

$${\rm{TEC}}=\frac{c({t}_{2}-{t}_{1})}{\kappa }\frac{{f}_{1}^{2}{f}_{2}^{2}}{{f}_{2}^{2}-{f}_{1}^{2}}$$

with the proportionality constant *κ* given by

$$\kappa =\frac{{c}^{2}{r}_{{\rm{e}}}}{2{\rm{\pi }}}\times {10}^{16}\approx 40.3\times {10}^{16}\,{{\rm{m}}}^{3}\,{{\rm{s}}}^{-2}$$

where *c* is the speed of light and *r*_e_ is the classical electron radius. Typical TEC values can be up to 200 TECU. Our analysis shows that the noise in the raw phone measurements has a standard deviation of around 70 TECU, more than 30 times larger than the measurement noise for traditional monitoring stations. Carrier-phase smoothing^19^ would ordinarily reduce the noise further (to 0.2 TECU (ref. ^20^)) but carrier-phase measurements are often unreliable on phones. Figure 2 compares the TEC measured by a particular monitoring station with the measurements from 1,011 nearby phones. Despite the far larger noise in individual phone measurements, when averaged they agree with the station measurements.

TEC measures the integral of the electron density along the straight-line path between a satellite and a phone. We adopt a standard thin-shell model of the ionosphere at a height of 350 km. The cosine of the angle between the line of sight and the shell normal converts between slant TEC (STEC) and vertical TEC (VTEC), the TEC for a path straight upwards. We partition the surface of the ionosphere into approximately 98,000 cells of roughly equal area (about 75 km edge length) using the S2 geometry library^21^ and assume that the VTEC is constant within each cell.

The hardware in satellites and mobile phones introduces different latencies when processing signals of different frequencies, leading to the so-called differential code bias (DCB). The biases shift the measured STEC from its true value as follows:

$${{\rm{STEC}}}_{{\rm{measured}}}={{\rm{STEC}}}_{{\rm{true}}}+{{\rm{DCB}}}_{{\rm{satellite}}}+{{\rm{DCB}}}_{{\rm{phone}}}$$

Although DCB values for satellites are published^22^, we must calculate the DCB for each individual phone. Substituting STEC = VTEC/cos(*θ*), where *θ* is the angle of incidence at the ionosphere piercing point, and rearranging, we obtain a linear equation for each line-of-sight measurement:

$$\frac{1}{\cos (\theta )}{{\rm{VTEC}}}_{{\rm{true}}}+{{\rm{DCB}}}_{{\rm{phone}}}={{\rm{STEC}}}_{{\rm{measured}}}-{{\rm{DCB}}}_{{\rm{satellite}}}$$

**Extended Data Fig. 1:**
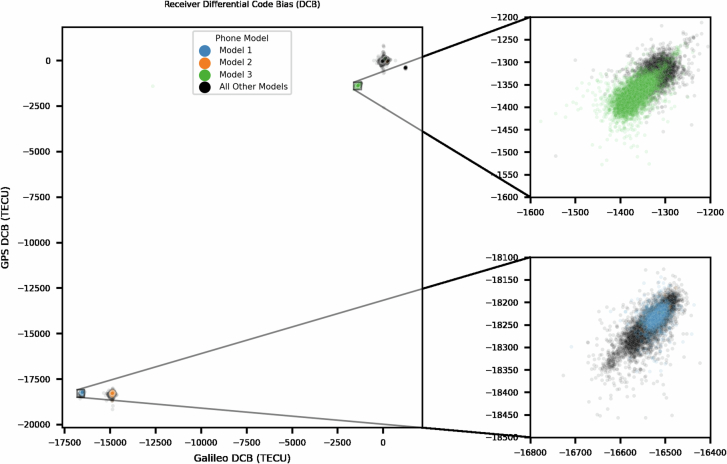
Estimated differential code bias clusters by phone model. The dots show the estimated differential code bias (DCB) for a sample of approximately 200,000 different phones from more than 800 different models. Values for three example models are colored to demonstrate our finding that phones of the same model have a similar DCB. A receiver can have a different bias when measuring signals from different satellite constellations, so the GPS and Galileo DCB are estimated separately. These are for the L1/L5 frequency pair that we use in this work. These sensor biases are far larger than the total electron content (TEC) being measured and must be accounted for. Source Data

Over 10-minute time windows, we solve for the unknown VTEC_true_ and DCB_phone_ using weighted least squares. In a typical time window, there are approximately 15,000–30,000 S2 cells with valid measurements and 100,000–500,000 phones. To make this tractable, we exploit the sparsity and block structure of the system (Methods). Our solutions show that DCB_phone_ clusters by phone model (Extended Data Fig. 1).

We evaluate our phone-based VTEC maps by comparing with the Madrigal database^23,24^. This database combines line-of-sight STEC measurements from over 9,000 monitoring stations for the US GPS and Russian GLONASS constellations at 30-second intervals, with carrier-phase smoothing to reduce noise. Our VTEC maps are spatially discretized into approximately 98,000 cells and temporally discretized into 1-minute windows. We compare our VTEC estimates in each cell-minute with measurements from the Madrigal database over the period of 10 September to 6 November 2023 to assess the accuracy and coverage of our measurements.

**Fig. 3:**
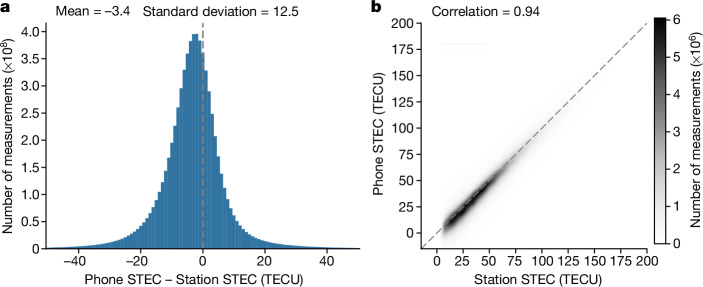
Quantitative comparison of monitoring station measurements with the ionospheric TEC derived from phone measurements. We validated against STEC measurements from monitoring stations in the Madrigal dataset from 10 September to 6 November 2023. For each measurement of the STEC along the path between a station and the satellite, we calculated the corresponding STEC from the phone-based VTEC map (see Methods for details). **a**, The differences between each station measurement and the phone-derived STEC. **b**, Comparison of Madrigal’s reported STEC with the STEC predicted by phone-based maps, revealing a Pearson correlation of 0.94. Owing to the large number of data points (7 billion STEC measurements), STEC values are discretized to the nearest TECU and plotted as a density. The axis ranges are limited to drop outliers while keeping 99% of the data points. The complete dataset is available in the Source Data. Station data in both plots were taken from the Madrigal database^23^. Phone data in both cases are from the present study. Source Data

When a part of the ionosphere is simultaneously measured by both stations and phones, we can compare the STEC implied by phone measurements with the STEC measured by the monitoring stations (Fig. 3; details in Methods). The phone-based TEC agrees well with the station measurements (Pearson correlation of 0.94), with the average phone-based TEC slightly lower than the Madrigal station measurements (mean offset of −3.4 TECU). Small offsets of this magnitude are typically found between published ionosphere models^25,26^ (see discussion in Methods).

**Extended Data Fig. 2:**
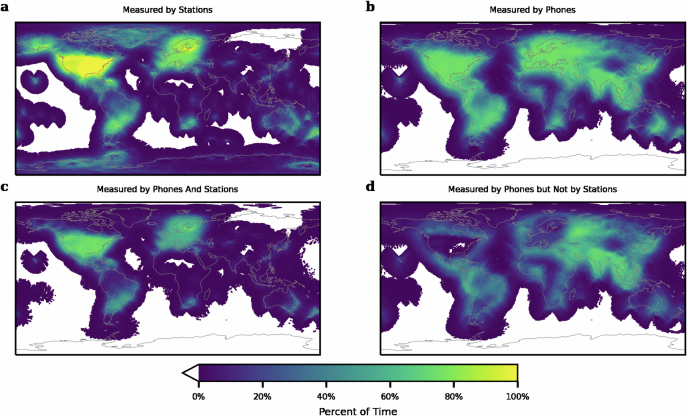
Spatial variation of ionosphere coverage for monitoring stations and phone measurement. The amount of time each part of the ionosphere is measured varies over the course of a day depending on the location of receivers on the ground and the orbit of satellites. The color indicates the percent of the time each part of the ionosphere is measured, based on the piercing point locations of measurements from each source. The coverage when using station measurements in the Madrigal database in (**a**) shows that the high density of stations in some parts of the world (e.g. the USA and Western Europe) leads to much higher coverage there than in other parts of the world. The corresponding map for phones in (**b**) indicates the percent of time that our ionosphere maps are able to estimate the vertical total electron content (VTEC) at each location. The coverage map for phones has a maximum of 78% of time which would increase if nighttime phone measurements were used. Moments where both stations and phones measure the same part of the ionosphere simultaneously allow quantitative comparisons to be made (see Fig. 3) and how often this happens in each place is shown in (**c**). The map in (**d**) shows the percent of time where each part of the ionosphere was measured by phones but **not** by monitoring stations. This indicates the places where our approach improves upon what was previously available (e.g. Asia, Africa, Eastern Europe, and South America). Plots were produced using all 30 second intervals over the 58 days from 2023-09-10 to 2023-11-06. Station data in all four plots were taken from the Madrigal database^23^ while all phone data are from the present study. Source Data

To compare the ionosphere measurement coverage between stations and phones, we compute the proportion of cell-minutes containing a value for the Madrigal database and for phone-based TEC maps. Over large portions of the globe (particularly Asia, Africa, Eastern Europe and South America), phones provide measurements of the ionospheric TEC at times and places missed by monitoring stations (Extended Data Fig. 2). Over the entire time period, 14% of the ionosphere was measured by stations alone, while 21% was measured by phones alone. Including phones with monitoring stations leads to measurements of 28% of the ionosphere, doubling the coverage of the ionosphere from monitoring stations alone.

The TEC maps from phone measurements capture the day–night cycle, with the peak in ionization in the early afternoon, and also show the Ionosphere Equatorial Anomaly, a crest of ionization north and south of the geomagnetic equator with a characteristic dip southwards over South America (Supplementary Videos 1 and 3).

**Fig. 4:**
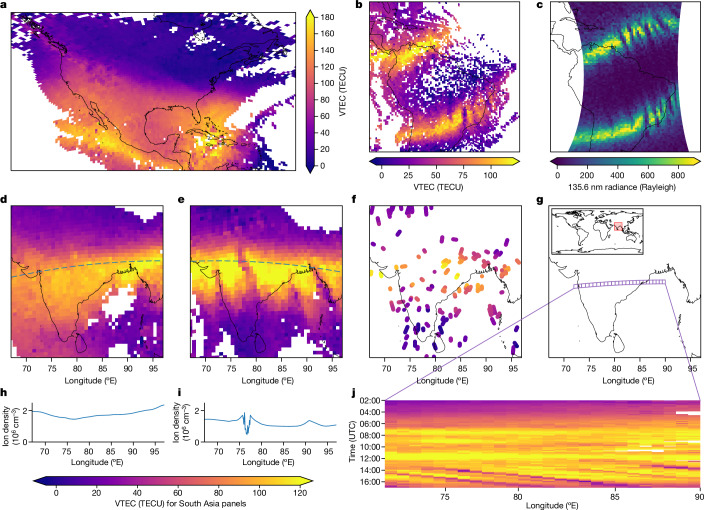
Ionospheric TEC features seen by phones and comparisons with other instruments. **a**, Ionospheric VTEC from phones during a geomagnetic storm (Kp index reaching 9) at 23:30 on 10 May 2024 showing the storm-enhanced density over North America. **b**,**c**, Plasma bubbles in the equatorial anomaly over South America at 00:20 on 13 October 2023 in ionospheric VTEC from phones (**b**) and in a far-ultraviolet image (**c**) of O i emission at 135.6 nm from the ionosphere F region captured by a geostationary satellite at 0.04-nm bandwidth (data from NASA/GOLD^37^). **d**–**j** Plasma bubbles over South Asia on 14 October 2023. Longitudinal features are visible in the equatorial anomaly in the ionospheric VTEC from phones at 14:39 (**e**) but were absent earlier at 12:57 (**d**). The dotted lines show the trajectory of a COSMIC-2 satellite measuring the local ion density at 520 km altitude (data from the University Corporation for Atmospheric Research (UCAR) COSMIC Program^29^). The ion density varies smoothly in **h** before the plasma bubbles have formed but shows a depletion in **i** as it passes through one of the bubbles. South Asia has few publicly available ionospheric monitoring stations so the VTEC measurements from the 18 monitoring stations (data from Madrigal^23^) shown in **f** fail to capture the detail seen by around 32,000 phones in **e** over the same time window. To visualize the time evolution of the longitudinal features, a slice of cells was chosen (**g**) and the VTEC measured by phones for the slice over time is shown in **j**. As the Sun rises, the ionization increases and the northern equatorial anomaly appears over this region. Near sunset, a series of longitudinal features appear in the equatorial anomaly moving eastwards at about 100 m s^−1^. Their motion can be seen as stripes from 14:00 onwards. All times are in UTC. Source Data

Solar storms affect the ionosphere, and we see signatures of such storms in our measurements. On 10–11 May 2024, an extreme G5 geomagnetic storm hit Earth causing visible auroras at lower latitudes. This storm, the biggest in more than 20 years, caused a strong disturbance in Earth’s magnetic field, with a Kp (planetary Kennziffer) index exceeding 9 reported by the National Oceanic and Atmospheric Administration’s Space Weather Prediction Center. Figure 4a shows the ionosphere measured by phones during this storm, revealing a cluster of TEC enhancement over the Caribbean and an intense, narrow plume of ionization often referred to as storm-enhanced density^27,28^ over North America. Supplementary Video 4 shows the intense TEC enhancement during the storm and the depletion afterwards.

**Extended Data Fig. 3:**
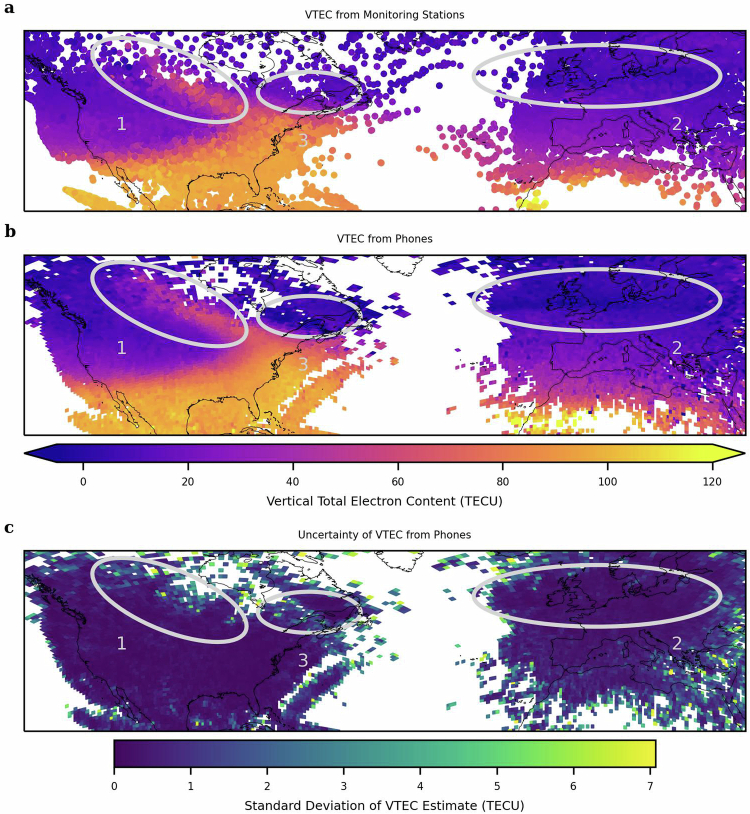
Comparison of features in the ionosphere VTEC caused by a geomagnetic storm. Measurements by 4,097 monitoring stations from the Madrigal database (**a**) and around 180,000 phones in our study (**b**). A strong geomagnetic storm on November 5th, 2023, 20:00 UTC (Kp index of 7) caused (1) storm-enhanced density over North America and (2) a mid-latitude ionospheric trough over Europe. In region (3) the VTEC estimated by phones is negative (non-physical) and disagrees with the VTEC from stations. In region (3), the configuration of satellites and receivers led to this part of the ionosphere getting few measurements, and so the vertical total electron content (VTEC) estimate from phones is poorly constrained as indicated by the large standard deviation in map (**c**). Monitoring station data in (**a**) were taken from the Madrigal database^23^. Phone data in (**b**) and (**c**) are from the present study. Source Data

**Extended Data Fig. 4:**
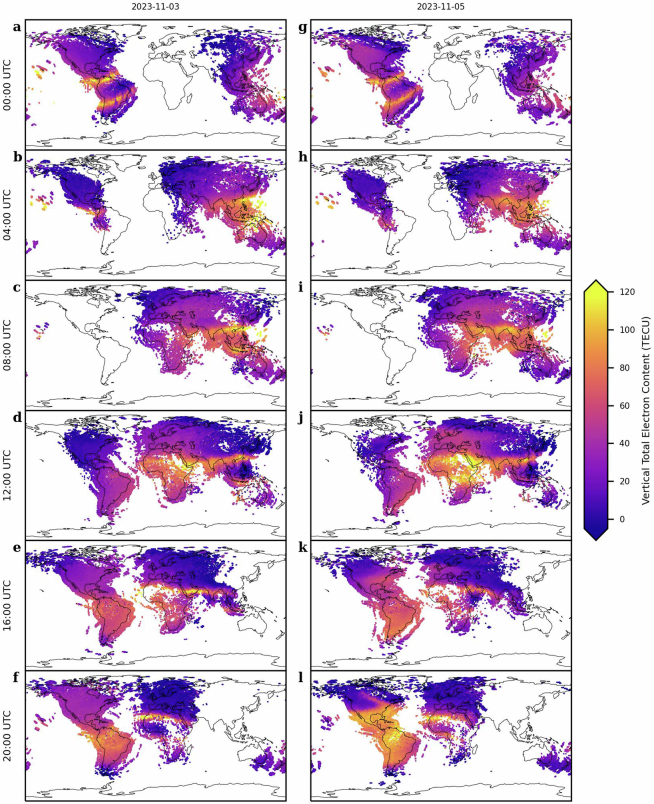
Comparison of ionospheric vertical total electron content (VTEC) measured by phones on two different days at the same times of day. **g-l** shows November 5th, 2023 when a geomagnetic storm caused storm enhanced density and a mid-latitude ionosphere trough. **a-f** shows November 3rd, 2023, a calm day before the storm hit. Source Data

Storm-enhanced density over North America was also seen in another geomagnetic storm on 5 November 2023. This time, phone measurements also reveal a depletion in ionization over Europe, a so-called mid-latitude ionospheric trough. Extended Data Fig. 3 compares maps from 180,000 phones and 4,097 monitoring stations. There is a striking contrast between phone measurements during the storm and the previous day’s measurements (Extended Data Fig. 4).

The phone measurements significantly extend the coverage of high-resolution TEC maps, making it possible to see interesting phenomena in parts of the world with few monitoring stations. Figure 4d–j shows longitudinal structures within the equatorial anomaly near sunset over South Asia that are not captured by the few monitoring stations available in that area. These structures lasted several hours and moved eastwards at around 100 m s^−1^ (Supplementary Video 2). We validate the observations using a COSMIC-2 (Constellation Observing System for Meteorology, Ionosphere, and Climate-2) satellite^29^ that passed through this structure at the same time, confirming a dip in ion density. The phone observations also match the characteristics (speed, tilt, width and spacing) of plasma bubbles observed over India using a nighttime all-sky imager during a campaign^30^ in 2008.

**Extended Data Fig. 5:**
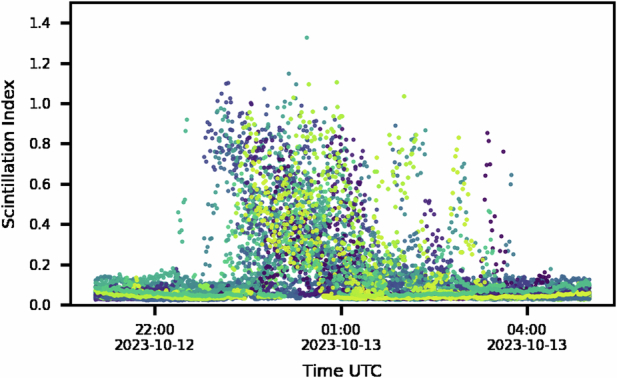
Scintillation during plasma bubbles over Brazil. Amplitude scintillation index (S4) computed from global navigation satellite system (GNSS) observables generated by a GNSS monitoring station deployed by the University of Colorado Boulder in São Paulo, Brazil. The measurement is obtained during the time and in an area that the ionosphere map from phone measurements observed plasma structures over South America (Fig. 4b,c). Calculating the scintillation indices requires high-rate measurements (10-100 Hz) in a carrier tracking loop which is beyond the capabilities of phone GNSS receivers. Scintillation refers to the rapid fluctuation in the amplitude and/or phase of the radio signal traversing plasma irregularities^2^. The S4 index is a measure of the magnitude of the signal amplitude fluctuation. Each color corresponds to measurements from a different satellite. At its peak, 15 of the 25 satellite measurements had an S4 index greater than 0.3. Scintillation degrades satellite navigation performance in the vicinity. Source Data

We also observed these longitudinal structures in the equatorial anomaly over South America. These phenomena have been recently observed in nighttime extreme-ultraviolet images from the geostationary GOLD (Global-scale Observations of the Limb and Disk) satellite^31^. The ionospheric VTEC measured by phones (Fig. 4b) agrees with the observations made by GOLD (Fig. 4c). The plasma bubble structures are telltale signs of plasma instability, which can result in scintillation of GNSS signals^4,32^ and disrupted radio navigation and communication systems^2^. Extended Data Fig. 5 shows that a GNSS monitoring station near São Paulo, Brazil, experienced strong scintillation during this time.

**Extended Data Fig. 6:**
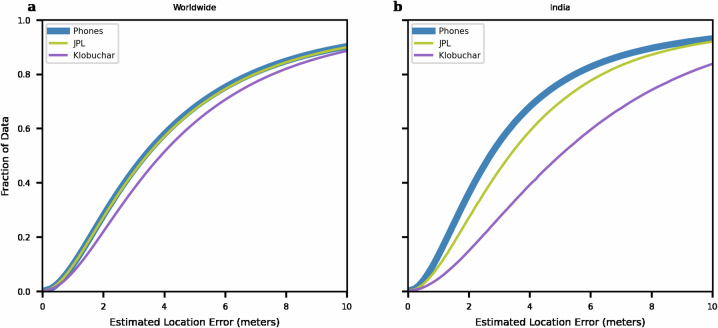
Estimated location error using different ionosphere models. The horizontal error is estimated using the Horizontal Dilution of Precision technique on the measurements from a held out set (0.4%) of global phones over the 36 days from October 17th to November 21, 2023. When the ionosphere corrections come from the model fit on phone measurements, the performance is similar to the JPL model when averaged globally (**a**) (Worldwide median: phones = 3.4 m, JPL = 3.4 m, Klobuchar = 3.9 m). When validated only on phones in India (**b**) the performance of the phone-based model is best (India median: phones = 2.7 m, JPL = 3.3 m, Klobuchar = 5.0 m). In this part of the world there are few publicly available monitoring station measurements to fit an ionosphere model but there is a high density of phone measurements. See Extended Data Fig. 7 to see this represented on a map. Phone data are from this study. JPL^3^ and Klobuchar^7^ model parameters were obtained from NASA CDDIS^36^. Source Data

**Extended Data Fig. 7:**
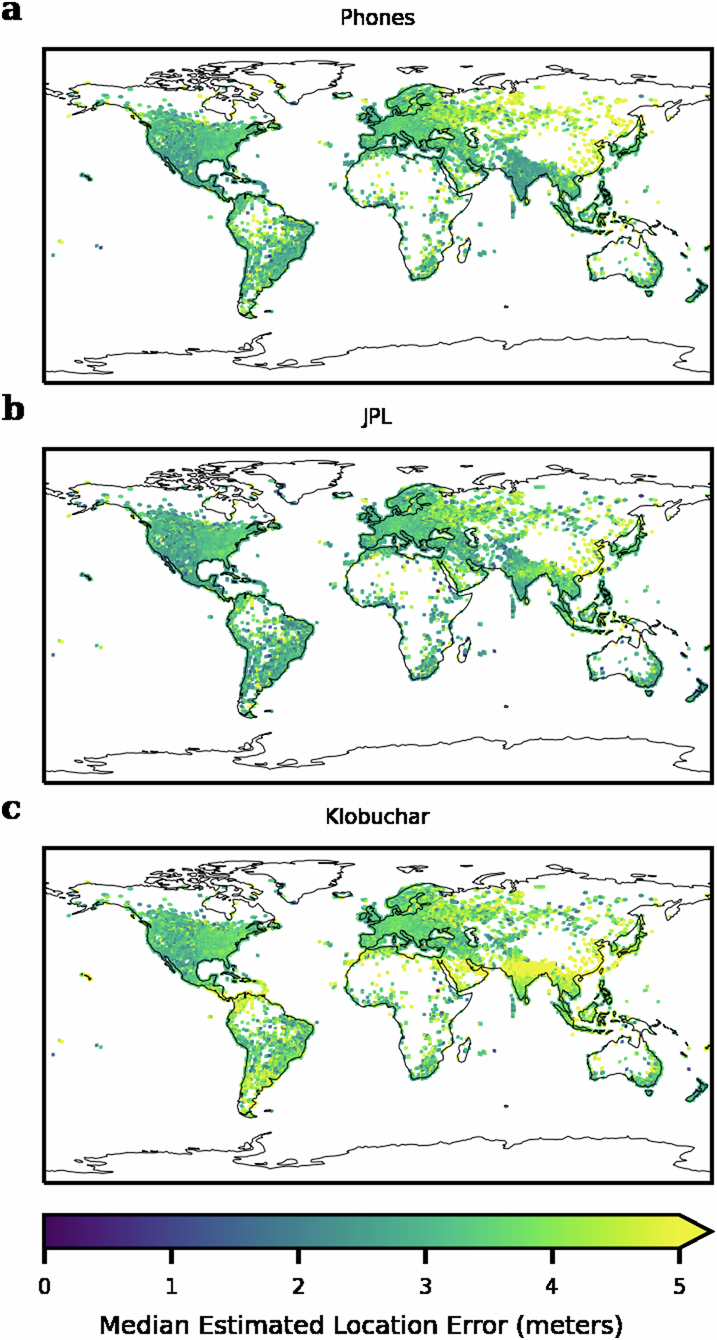
Geographical variation of estimated location error. The horizontal error is estimated using the Horizontal Dilution of Precision technique on the measurements from a held out set (0.4%) of global phones over the 36 days from October 17th to November 21, 2023. The performance of the ionosphere model from phones (**a**) is similar to the JPL model^3^ (**b**) in most places but it gets lower error in India where the ionosphere is quite dynamic and there are many phones but few monitoring stations. Both perform better than the Klobuchar model^7^ used in most phones today (**c**). Phone data are from this study. JPL and Klobuchar model parameters were obtained from NASA CDDIS^36^. Source Data

Mitigating ionospheric error in satellite-based positioning for Android users is the primary aim of this work. The increased detail and coverage of phone-based TEC maps leads to improved location accuracy, particularly in parts of the world with few monitoring stations (Extended Data Figs. 6 and 7 and Methods). Efforts are underway to deliver the location-accuracy improvements enabled by this research to Android users.

Although measurements from individual mobile phones are noisier than those from conventional monitoring stations, we have shown that millions of phones in concert yield valuable measurements of ionospheric TEC. Other recent work has shown that phone accelerometers can detect earthquakes to provide early warning^33^ and that phone barometers can improve weather forecasting^34^. Building on these examples, our work continues to illuminate the potential for mobile phone sensors as a powerful tool to improve the scientific understanding of our planet.

## Methods

### Data collection and outlier rejection

The phone measurements used in this work are from a population of Android devices that have location and relevant settings enabled and are using satellite signals to determine location. Android periodically collects sensor measurement data from this population of Android devices to provide and improve location-based services. This collection is limited to conserve battery, memory and network use. More information about Android’s collection and use of location data is available at https://policies.google.com/technologies/location-data.

Our research used the collected measurements to make ionospheric TEC maps that can improve location accuracy. For this feasibility study, we used a subset of the collection with daytime satellite measurements (05:00–22:00 local time) and latency up to several days. For simplicity, we limited our analysis to measurements of US GPS and European Galileo constellations at frequencies of 1,575 MHz and 1,176 MHz (known as L1 and L5), accounting for 70% of dual-frequency phone measurements. With these restrictions, we used measurements from between 200,000 and 2 million unique phones per hour, significantly larger than the approximately 9,000 monitoring stations available in the Madrigal database. Our dataset included measurements from about 40 million dual-frequency phones each day.

TEC is a measure of the integral of free-electron density along the straight-line path between a satellite and a phone. We adopted a standard thin-shell model of the ionosphere at a height of 350 km above Earth, allowing us to combine measurements along different paths based on where they intersect the shell, the so-called ionosphere piercing point.

Determining the path taken by the radio signal through the ionosphere requires positions of both the satellite and the phone. Whereas accurate satellite positions are available from published orbit parameters, phone position is calculated by the GNSS receiver in the phone. To maintain user privacy, we used only the location of each phone coarsened to an approximately 10-km grid, and we removed isolated phones with locations far from populated areas. Although this limits the resolution of features in our calculated TEC map, we see that this is still sufficient for resolving the small-scale ionospheric features necessary for location-accuracy improvement and for observations of scientific phenomena.

Although dedicated GNSS receivers reduce the noise in the calculated TEC using an additional measurement of the carrier phase^19^, this carrier phase is often unavailable or too noisy in phone receivers. Instead, measurements were collected with a frequency of up to 1 Hz and were aggregated using an uncertainty weighted average over a 1-minute window. Time windows with fewer than ten measurements were dropped.

When solving the linear system to yield the ionospheric VTEC in each cell and the DCB of each phone, some receivers or ionosphere cells are poorly constrained. These phones and cells were removed so that the calculation could proceed without regularization. The calculation is also sensitive to outlier measurements so we filtered these in advance by removing measurements more than 300 TECU from the median for that phone and constellation (about 0.4% of the phone measurements).

### Exploiting blockwise structure

Estimating VTEC requires solving a system of linear equations of the form

$$\frac{1}{\cos (\theta )}{{\rm{VTEC}}}_{{\rm{true}}}+{{\rm{DCB}}}_{{\rm{phone}}}={{\rm{STEC}}}_{{\rm{measured}}}-{{\rm{DCB}}}_{{\rm{satellite}}}$$

where VTEC_true_ and DCB_phone_ are unknown. As each phone has a unique bias for each satellite constellation, we must jointly fit thousands of ionospheric parameters and millions of bias terms. We do this by minimizing the squared error:

$${x}^{\ast }=\mathop{{\rm{a}}{\rm{r}}{\rm{g}}{\rm{m}}{\rm{i}}{\rm{n}}}\limits_{x}{\Vert Mx-y\Vert }_{2}^{2}$$

where *y* *=* STEC_measured_ − DCB_satellite_, *x* is a vector containing both ionospheric parameters and phone bias values, and *M* encodes the linear relationship between these quantities. In this case, *M* is a sparse matrix and takes the form

$$M=\,\left[\begin{array}{cc}R & S\end{array}\right]$$

The matrix *R* has shape *n* × *r*, where *n* is the number of measurements and *r* is the number of ionospheric parameters—in this case, corresponding to tens of thousands of S2 cells. The matrix *S* has shape *n* × *s*, where *s* is equal to the number of (phone, constellation) pairs in the dataset, as each phone has a unique bias for each constellation.

Solving the least-squares problem amounts to exactly solving the linear system

$${M}^{T}{Mx}={M}^{T}y$$

where *T* indicates matrix transpose. Using the structure described above, we have

$${M}^{T}M=\left[\begin{array}{cc}A & B\\ {B}^{T} & D\end{array}\right]=\left[\begin{array}{cc}{R}^{T}R & {R}^{T}S\\ {S}^{T}R & {S}^{T}S\end{array}\right]$$

*A* has the shape *r* × *r*, where *r* is on the order of tens of thousands. It is sparse, having non-zero entry in position *ij* only when there is a receiver that sees both S2 cells *i* and *j*. In this sense, it is a kind of incidence matrix between S2 cells. In particular, *A* is tractable to invert and apply.

The matrix *B* is a weighted incidence matrix between S2 cells and receivers with shape *r* × *s*, and is short and wide.

*D* has shape *s* × *s*, where *s* is potentially in the millions. However, *S* has only one non-zero entry per line, corresponding to the bias of the (receiver, constellation) pair for the particular measurement. As a result, *D* is diagonal and can be inverted and applied trivially.

We can now apply the blockwise matrix inversion identity

$${({M}^{T}M)}^{-1}=\left[\begin{array}{cc}{(A-B{D}^{-1}{B}^{T})}^{-1} & -{(A-B{D}^{-1}{B}^{T})}^{-1}B{D}^{-1}\\ -{D}^{-1}{B}^{T}{(A-B{D}^{-1}{B}^{T})}^{-1} & {D}^{-1}+{D}^{-1}{B}^{T}{(A-B{D}^{-1}{B}^{T})}^{-1}B{D}^{-1}\end{array}\right]$$

If we are interested only in extracting ionospheric parameters and not the biases for each individual receiver, then we need only consider the terms from the first row of this equation and we get

$${x}_{\text{VTEC}}={(A-B{D}^{-1}{B}^{T})}^{-1}[\begin{array}{c}I-B{D}^{-1}\end{array}]{M}^{T}y$$

*P* = *A* − *BD*^−1^*B*^*T*^ is known as the Schur complement of *D*. It has size *r* × *r* and is tractable to invert, whereas *D* is diagonal and is trivial to invert.

In addition to solving for the maximum likelihood value for each coefficient, we would also like to estimate the variance associated with each coefficient. The variance of the solution *x* is given by

$$\mathrm{cov}(x)={({M}^{T}M)}^{-1}$$

As we are only interested in the covariance of the ionosphere coefficients, we can take the upper left portion of this covariance matrix:

$$\mathrm{cov}({x}_{\text{VTEC}})={P}^{-1}={(A-B{D}^{-1}{B}^{T})}^{-1}$$

In particular, we are interested in the diagonal elements, as these are the variances of the individual coefficients. However, this is still a large sparse matrix and we would like to avoid materializing it and inverting it directly just to obtain the diagonal. Instead, we use an unbiased estimator for the diagonal of a matrix:

$$d=v\,\ast \,{P}^{-1}v$$

where *v* is a vector-valued random variable whose entries are drawn uniformly from {+1, −1}, and the asterisk denotes entry-wise multiplication. This approximation generalizes the well-known Hutchinson trace estimator^38^. To see that this is an unbiased estimator, we first note that

$${\mathbb{E}}({v}_{j}{v}_{k})=\{\begin{array}{cc}1 & {\rm{if}}\,j=k\\ 0 & {\rm{if}}\,j\ne k\end{array}$$

We can now compute the expectation of the *j*th element of the estimator:

$$\begin{array}{l}{\mathbb{E}}({d}_{j})\,=\,{\mathbb{E}}(\sum _{k}{P}_{jk}^{-1}{v}_{j}{v}_{k})\\ \,\,=\,\sum _{k}{P}_{jk}^{-1}{\mathbb{E}}({v}_{j}{v}_{k})\\ \,\,=\,{P}_{jj}^{-1}\end{array}$$

as required. To reduce the variance of this estimate, we apply the procedure to a large number of randomly sampled vectors *v* and take the average. This allows the ionospheric VTEC and the uncertainty to be estimated from a large number of phone measurements efficiently.

### Comparison with monitoring station measurements

Phone measurements and monitoring stations generally agree, but disagreements also exist and are discussed in this section. We compared with monitoring station line-of-sight STEC measurements from the Madrigal database^23^. We computed the phone-based STEC estimate for each line of sight from the map using the VTEC at the piercing point and the slant angle to convert to STEC.

One possible source of disagreements is the conversion of the phone-generated VTEC to STEC. This conversion process assumes that the ionosphere is a two-dimensional thin shell at 350 km altitude. This modelling assumption allows the STEC to be computed for some lines of sight not measured by phones if the VTEC at the piercing point has been measured along a different line of sight. The Madrigal dataset also applies a thin-shell assumption (for bias calibration), but there may be small differences in how Earth’s curvature and electron-density profiles are accounted for when applying the zenith-angle correction to convert STEC to VTEC.

In some places and times, the phone-based VTEC estimate is particularly uncertain. Phone measurements are noisy, so a large number must be averaged to get a reliable estimate. In some cases, averaging a few noisy measurements results in a negative (non-physical) VTEC estimate. In addition, the linear system for VTEC and receiver DCBs is poorly constrained when the baselines separating receivers on the ground are short, leading to piercing points being measured at a narrow range of slant angles. Maps can also be poorly constrained when few receivers are making measurements to few satellites. These poorly constrained regions shift over time as the geometry of satellites and receivers changes. Fortunately, our method also provides an uncertainty for each VTEC estimate, and these situations can be distinguished by their large uncertainty. VTEC estimates with standard deviation larger than √50 TECU are excluded from the maps. Extended Data Fig. 3 shows an example of a disagreement between phone-based maps and station measurements with high uncertainty in the phone-based map, indicating that the phone-based map is unreliable in this region.

GNSS measurements are sometimes biased by an effect known as multipath^19^. This occurs when the radio signal reaches the receiver by two or more paths and is commonly caused by reflections from the ground or buildings. Phones are more susceptible to this than monitoring stations owing to their complex operating environments. We tried removing multipath measurements but found that the detection methods were unreliable.

Any of these issues could explain the bias shown in Fig. 3 for the quantitative comparison of monitoring station measurements with the ionosphere TEC derived from phone measurements. Similar magnitudes of TEC bias have also been observed in other measurement methods, such as lightning and satellite altimetry, and among the various global ionosphere models^25,26^.

### Location-accuracy improvement

The primary aim of this work was to improve the location accuracy for Android users by providing more accurate corrections for ionospheric effects. We evaluated the difference in the horizontal location accuracy when different ionospheric models are used by applying the dilution of precision technique^39^ on a held-out set of phones. To form a baseline for comparison, we also evaluated two published TEC models: a global ionospheric TEC model from the NASA Jet Propulsion Laboratory (JPL)^3^ and the Klobuchar model^7^.

The Klobuchar model was developed in the early days of GPS and was optimized for the limited computation and bandwidth available at the time. It uses only eight parameters that are broadcast by the satellite and updated daily. The Klobuchar model is still used in the majority of mobile phones, because it is free, reliably available in real time and easy to use. It is sometimes tightly integrated into GNSS hardware in phones.

The JPL Final model has a latency of approximately 3 days, incorporating measurements from 200 monitoring stations to produce a global map of VTEC for every 2 hours on a grid of 2.5° latitude by 5° longitude using a Kalman filter. This model demonstrates the performance when monitoring station measurements are carefully applied.

Extended Data Fig. 6 shows that when evaluated globally, the TEC model fit on phone measurements provides a similar performance to the JPL model and improved performance compared with the Klobuchar model. When limited to India, where there are far more phone measurements available than monitoring stations, the phone-based model outperforms both the Klobuchar model and the JPL model. Extended Data Fig. 7 shows the spatial distribution of location error, confirming this finding in regions of the world with sparse networks of monitoring stations.

### Instruments for ionosphere observation

GNSS receivers on the ground are not the only way to observe the ionosphere. Orbiting satellites also use GNSS receivers to track their position, and the same technique can be used to measure the TEC between low-Earth-orbit (LEO) satellites and navigation satellites. Orbit determination receivers map the TEC above LEO satellite orbits^40^. Radio occultation measures the TEC at opportune moments when radio signal paths pass through the limb of the atmosphere and enter side-looking antennas onboard LEO satellites^41^. These LEO satellites may also capture GNSS signals reflected off calm waters or ice on Earth’s surface through GNSS reflectometry techniques to measure the TEC along the reflection signal path^42^. All-sky imagers map the emission of ions and neutral particles in the ionosphere at night and reveal structures in the varying plasma density, both from the ground^43,44^ and in space^45–47^. Incoherent scatter radars^48^, coherent radars^49^ and ionosondes^50^ transmit radio waves and record the incoherent backscatters and coherent reflections from layers in the ionosphere to profile the ionospheric electron-density distributions in the radar field of view. In situ measurements can also be performed by LEO satellites such as COSMIC-2 and Swarm, which measure the plasma density along a trajectory inside the ionosphere^51,52^.

## Online content

Any methods, additional references, Nature Portfolio reporting summaries, source data, extended data, supplementary information, acknowledgements, peer review information; details of author contributions and competing interests; and statements of data and code availability are available at 10.1038/s41586-024-08072-x.

## Supplementary information

Supplementary Video 1 Video of ionosphere TEC measured by phones—short, high resolution. The video shows the ionosphere VTEC measured by phones globally over a day. Each frame is an independent estimation of the VTEC using measurements from a 10-minute time window centred on the timestamp in UTC in the title. There are gaps in coverage where no phone measurements are available due to low population or disabled collection during nighttime. Short duration but higher resolution.

Supplementary Video 2 Animation of plasma bubbles over South Asia. This animation of ionosphere measurements from phones shows longitudinal features in the northern equatorial anomaly moving eastwards near sunset on 14 October 2023. These strong gradients in ionization degrade the accuracy of satellite-based navigation systems unless they can be compensated for using a detailed ionosphere map. The lack of phone measurement collection at night leads to the drop in coverage near the end of the animation.

Supplementary Video 3 Video of ionosphere TEC measured by phones—long, lower resolution. The video shows the ionosphere VTEC measured by phones globally over several weeks. Each frame is an independent estimation of the VTEC using measurements from a 10-minute time window centred on the timestamp in UTC in the title. There are gaps in coverage where no phone measurements are available due to low population or disabled collection during nighttime.

Supplementary Video 4 Video of May 2024 geomagnetic storm. On 10–11 May 2024, an extreme G5 geomagnetic storm, the strongest storm since the 2003 Halloween Solar Storm, occurred with the planetary Kp index reaching 9. This video shows some of the impact of this storm on the ionosphere captured by the TEC maps constructed from phone measurements. To capture the magnitude of the highly elevated TEC values, the video used a peak TEC scale of 200 TECU. Most other figures and videos presented in this paper used 120 TECU as the peak values. On 10 May, the usual South American equatorial anomaly or crests of ionizations were pushed apart to higher latitudes in both the Northern and Southern hemispheres. At around 20:00 UTC, the northern crest can be seen building up along the coast of Central America and subsequently stretching northwards into the western United States by 21:00 UTC. Streaks of ionization can be seen converging towards the high latitudes of North America’s western regions to form storm-enhanced density until 00:00 UTC the following day. By 22:00 UTC, a cluster of TEC enhancement formed over the Caribbean and Southeast United States and lingered over the area for over 4 hours until after 02:00 UTC on 11 May. The southern crest shifted downwards around 22:00 UTC. Enhanced TEC can be observed over Pacific Islands extending to Northern Australia during this time. By 03:00 UTC and onwards until 09:00 UTC on 11 May, the equatorial ionospheric anomalies extended northwards into China and southwards to Australia. Plasma structures continued to linger over Australia (and sometimes over the North Island of New Zealand) until 13:00 UTC. After this time, the ionosphere TEC remained well below its normal values. This is especially evident starting around 16:00 UTC over the Americas.

## Source data

Source Data Fig. 1

Source Data Fig. 2

Source Data Fig. 3

Source Data Fig. 4

Source Data Extended Data Fig. 1

Source Data Extended Data Fig. 2

Source Data Extended Data Fig. 3

Source Data Extended Data Fig. 4

Source Data Extended Data Fig. 5

Source Data Extended Data Fig. 6

Source Data Extended Data Fig. 7

## Data Availability

A dataset of ionosphere maps fit using phone measurements is available at 10.24433/CO.9149928.v1 (ref. ^53^). The accompanying video visualizes the dataset over time. Note that this dataset is too large to include as a supplementary video. For validation, we compare with several other instruments measuring the ionosphere. GOLD data courtesy of NASA/GOLD and the mission science team (https://gold.cs.ucf.edu/data/). COSMIC-2 data courtesy of UCAR COSMIC Program (10.5065/T353-C093). Madrigal line-of-sight measurements courtesy of MIT Haystack CEDAR database (http://cedar.openmadrigal.org/). Station measurements and station differential code bias estimates courtesy of International GNSS Service (https://igs.org/). Base maps use freely available data from https://www.naturalearthdata.com/downloads/. GPS TEC data products and access through the Madrigal distributed data system are provided to the community by the Massachusetts Institute of Technology under support from US National Science Foundation grant AGS-1952737. Data for the TEC processing is provided from the following organizations: UNAVCO, Scripps Orbit and Permanent Array Center, Institut Geographique National, France, International GNSS Service, The Crustal Dynamics Data Information System (CDDIS), National Geodetic Survey, Instituto Brasileiro de Geografia e Estatística, RAMSAC CORS of Instituto Geográfico Nacional de la República Argentina, Arecibo Observatory, Low-Latitude Ionospheric Sensor Network (LISN), Canadian High Arctic Ionospheric Network, Institute of Geology and Geophysics, Chinese Academy of Sciences, China Meteorology Administration, Centro di Ricerche Sismologiche, Système d’Observation du Niveau des Eaux Littorales (SONEL), RENAG: REseau NAtional GNSS permanent (10.15778/resif.rg), GeoNet - the official source of geological hazard information for New Zealand, Finnish Meteorological Institute, SWEPOS - Sweden, Hartebeesthoek Radio Astronomy Observatory, TrigNet Web Application, South Africa, Australian Space Weather Services, RETE INTEGRATA NAZIONALE GPS, Estonian Land Board, TU Delft, Western Canada Deformation Array, EUREF Permanent GNSS Network, GeoDAF: Geodetic Data Archiving Facility, African Geodetic Reference Frame (AFREF), Kartverket - Norwegian Mapping Authority, Geoscience Australia, IGS Data Center of Wuhan University, Pacific Northwest Geodetic Array, Nevada Geodetic Laboratory, Earth Observatory of Singapore, National Time and Frequency Standard Laboratory - Taiwan, and Korea Astronomy and Space Science Institute. Source data are provided with this paper.

## Code availability

A Python notebook is available at 10.24433/CO.9149928.v1 (ref. ^53^) containing the open source code used in this work to process the phone measurements into an ionosphere map and to visualize it.
