# Publisher Correction: Mapping the ionosphere with millions of phones

**DOI:** 10.1038/s41586-024-08520-8

**Published:** 2024-12-17

**Authors:** Jamie Smith, Anton Kast, Anton Geraschenko, Y. Jade Morton, Michael P. Brenner, Frank van Diggelen, Brian P. Williams

**Affiliations:** 1 Google Research, Mountain View, CA USA; 2 Aerospace Engineering Sciences Department, University of Colorado Boulder, Boulder, CO USA; 3 School of Engineering and Applied Sciences, Harvard University, Cambridge, MA USA; 4 Google Platforms and Devices, Mountain View, CA USA

**Keywords:** Space physics, Atmospheric dynamics, Technology

Correction to: *Nature* 10.1038/s41586-024-08072-x Published online 13 November 2024

In the version of the article initially published, there was an error in the Fig. 4b,c caption, now reading “Plasma bubbles in the equatorial anomaly over South America at 00:20 on 13 October 2023,” where 13 October 2023 appeared originally as “13 May 2024.” The error has been corrected in the HTML and PDF versions of the article.

