# Supplementary figures and images for: Mapping the ionosphere with millions of phones
Source: Nature. 2024 Nov 13;635(8038):365–9. doi: 10.1038/s41586-024-08072-x (PMC11560844; doi:10.1038/s41586-024-08072-x)

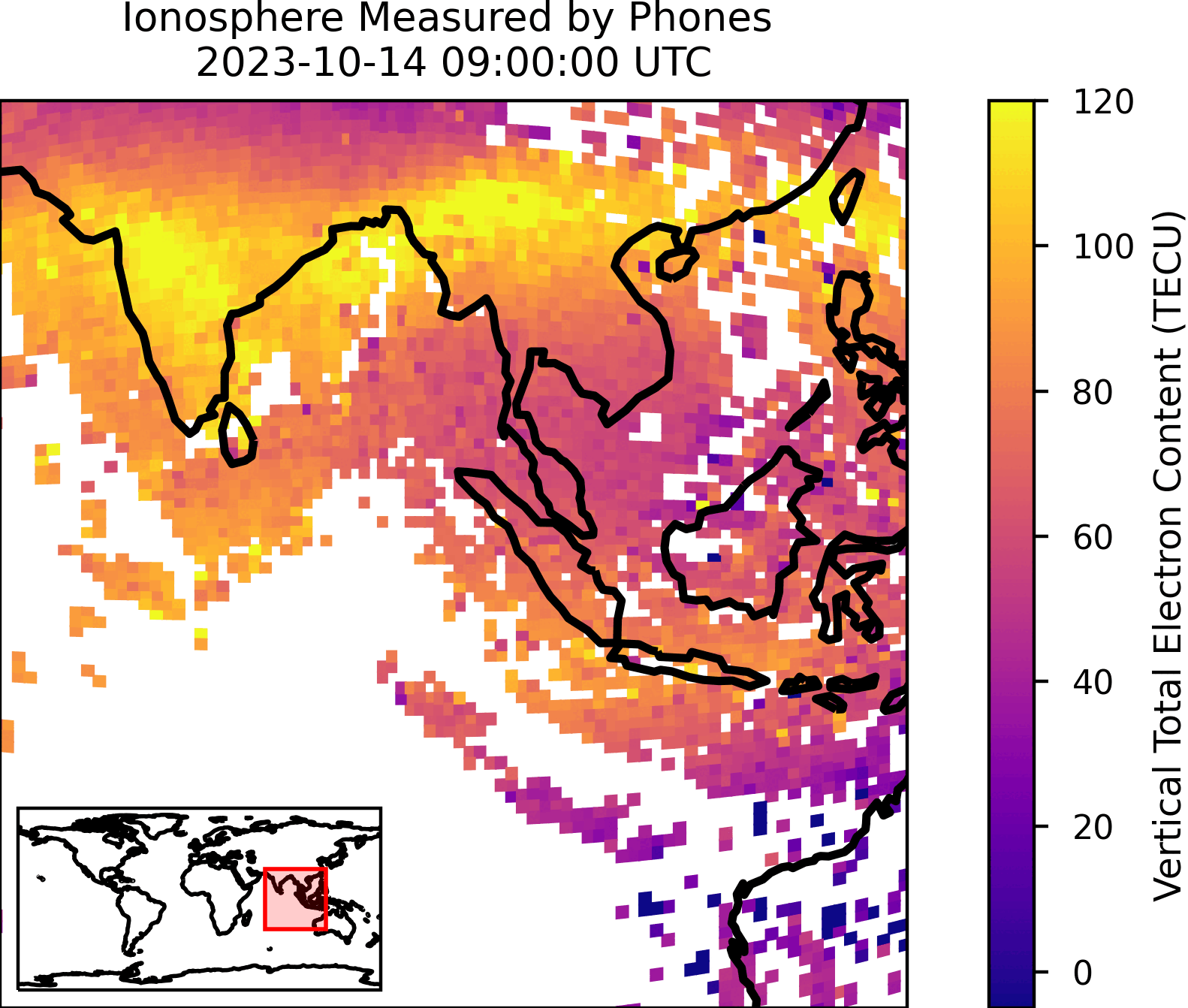

Supplement: Supplementary file 2 — Animation of plasma bubbles over South Asia. This animation of ionosphere measurements from phones shows longitudinal features in the northern equatorial anomaly moving eastwards near sunset on 14 October 2023. These strong gradients in ionization degrade the accuracy of satellite-based navigation systems unless they can be compensated for using a detailed ionosphere map. The lack of phone measurement collection at night leads to the drop in coverage near the end of the animation. [file 41586_2024_8072_MOESM2_ESM.gif]
